# Understanding Ethical, Legal and Societal Issues (ELSIs) in Human Biobanking and Genomics for Research and Healthcare in Zimbabwe: The Genomics Inheritance Law Ethics and Society (GILES) initiative

**DOI:** 10.12688/aasopenres.12917.2

**Published:** 2019-06-12

**Authors:** Alice Matimba, Andrew Chimatira, Oppah Kuguyo, James January, Zivayi Mupambireyi, Bazondlile Marimbe-Dube, Vasco Chikwasha, Zibusiso Nyati-Jokomo, Shamiso Muteti, Pedzisayi Mangezvo, Abigail Kangwende, Alfred Chingono, Midion Chidzonga, Jonathan Gandari, James Hakim, Kusum Nathoo, Christopher Samkange, Walter Mangezi, Sandra Lee, Lovemore Gwanzura, Mildred Cho, Paul Ndebele

**Affiliations:** 1 College of Health Sciences, University of Zimbabwe, Harare, Zimbabwe; 2 Centre for Sexual Health and HIV Research Zimbabwe (CeSHHAR Zimbabwe), Harare, Zimbabwe; 3 Faculty of Health Sciences, Africa University, Mutare, Zimbabwe; 4 Biomedical Ethics, Stanford University School of Medicine, Stanford, CA, USA; 5 Medical Research Council of Zimbabwe, Harare, Zimbabwe; 6 Milken Institute School of Public Health, George Washington University, Washington, DC, USA

**Keywords:** biobanking, human genomics, genomic research, ethics, ELSI, Zimbabwe, Africa

## Abstract

Biobanks and human genomics applications are key for understanding health, disease and heredity in Africa and globally. Growing interest in these technologies calls for strengthening relevant legal, ethical and policy systems to address knowledge disparities and ensure protection of society, while supporting advancement of science. In Zimbabwe there is limited understanding of ethical, legal, and societal issues (ELSI) for biobanking and genomics. The Genomics Inheritance Law Ethics and Society (GILES) initiative was established in 2015 to explore the current status and gaps in the ethical and legal frameworks, knowledge among various stakeholders, and to establish capacity for addressing ELSI of biobanking and genomics as applied in biomedical and population research, and healthcare. The project was conducted over a countrywide geographical region and established one of the most comprehensive studies for ELSI of human biobanking and genomics in Africa. This paper outlines the strategy undertaken during the implementation of the GILES initiative and discusses the importance of such an initiative for characterisation of ELSI of human biobanking and genomics in Zimbabwe and Africa.

## Introduction

Biobanks of human biospecimen collections are key resources for understanding individual and population diversity, and are integral to healthcare research, medical care, and drug discovery ^1, 2^. Linked to biobanking, advances in technology are enabling large-scale biochemical and genomic analysis, generating substantial amounts of data of personal and health relevance with ethical implications for communities and populations ^3– 6^. Although the benefits of human biobanking and genomics applications are well recognised, ethical, legal and social challenges arise alongside unclear regulations and policies, and limited understanding among research scientists, healthcare professionals and the wider public ^7^. In particular, African countries are faced with a growing need for the application of genomics in medicine and research. African genomics and population data are drawing regional and global interests as they add rich genomic background diversity to existing efforts to fully understand human genomic variation. This plays an important role in biomarker identification, improving disease diagnostics, and development of targeted therapies, which take into account interplay of environmental and demographic factors ^8^. However, the nature of biobanking and genomics gives rise to ethical and social issues at personal and population level. Therefore, there is urgent need to understand the current status, gaps and needs to build capacity for appropriately applying these technologies at the national, regional and international level. The main objective of this article is to describe the strategy and experiences of the Genomics Inheritance Law Ethics and Society (GILES) initiative aimed at understanding ethical, legal and societal issues (ELSIs) in human biobanking and genomics. In this article we build upon the rationale for addressing ELSIs in Africa using Zimbabwe as an example of a country with less advanced structures for human biobanking and genomics and where ELSIs are poorly understood among professional and community groups. We highlight challenges and opportunities observed during the implementation of the project and outline potential locally tailored approaches for comprehensive characterization and capacity building for ELSIs of human biobanking and genomics in Africa.

## The need for ELSI research for human biobanking and genomics in Africa

Recently, several consortia have embarked on projects to characterize African population genomics. The largest consortium is the Human Heredity and Health in Africa (H3Africa) program, which is focused on supporting biobanking and collaborative genomics research for understanding population genomic diversity in relation to disease susceptibility, diagnosis and association with environmental factors ^9– 11^. This and other ongoing initiatives create the need for anticipating and addressing emerging issues in human genomics notably: increased biobanking activities, whole genome sequencing, genome wide association studies, large scale databases and bioinformatics. Researchers in Zimbabwe are actively contributing to this initiative, and other related continent-wide consortia whereby associated ethical, legal, and societal issues (ELSI) remain under-explored. For example, despite the expectations of the international collaborative projects in the cross-border storage of human biospecimens and depositing of research results in consortia databases for access by scientists locally and abroad, differing terms and norms which are likely to present barriers to access and use are not well addressed. In addition, the uni-directional flow of samples and data out of Africa has created a sense of exploitation and distrust and the African genomics research community are playing a leading role in addressing such concerns and limitations as they become more likely to occur ^12, 13^.

## Biobanking, genomics and emerging ELSIs in Zimbabwe

In Zimbabwe, human biospecimen collections or biobanks have largely focused on infectious diseases research, national surveillance programs, disease outbreaks and molecular diagnostic applications. In this work, we acknowledge the existence of biobanks both in their rudimentary and advanced form, and the potential for their samples to be used for a wider variety of human genomics applications than for which they were originally collected. To date, the Biobank and Pharmacogenetics Database of African Populations is the only openly reported resource, which marked a significant step in multi-national collaborative biobanking efforts, and was designed for the study of variations associated with drug response in Africa ^14^. Such activities were established with limited knowledge and expertise about ELSIs and create a basis for strengthening the current structures for human biobanking and genomics sciences oversight.

Although biobanking and genomics are still in their infancy in Zimbabwe, growing interest and participation of local researchers in international collaborative consortia promises new avenues for research and medical solutions important to public health. For example, a local pharmacogenetics-based study indicated that the prescribed use of the anti-HIV drug efavirenz may result in severe side effects among patients due to highly prevalent variants in the gene encoding the drug metabolising *CYP2B6*, which were associated with decreased drug clearance, and thereby increasing risk of side effects such as depression and other neuropsychiatric complications ^15– 17^. Clinical trials to assess the possibility of reducing dose and cost-effectiveness of pharmacogenetics-based prescriptions are underway. The benefit of such examples of translational research cannot be underestimated and more clinical research involving biobanking and genomics is highly anticipated in the near future. As more awareness builds among researchers, healthcare professionals and policymakers, the applications of biobanking, genomics research and bioinformatics will increase bringing to light the deficiencies in the current ELSI framework in Zimbabwe.

In the wider community, individual and society beliefs, practices and perceptions influence participation in biospecimen collection for human genomics. As with most African countries, Zimbabwe is undergoing socio-economic and cultural as well as religious transitions, which impacts on beliefs and practices towards health research involving biobanking. In Africa, blood sample collection is a major area of concern among community and religious groups, and may be viewed by many as part of “witchcraft” ^18– 20^.

Zimbabwe is a landlocked country in the Southern African region with a population of approximately 15 million inhabitants. Being centrally located, Zimbabwe provides a major link for trade and migration, and access to a diverse ethnic and highly literate population ^21^. While Zimbabwe is undergoing constitutional reforms, scientific and technological advances, protection of researchers and participants may become more compromised. Ethico-legal consequences and risks of psychosocial harm, stigma and genetic discrimination also need to be addressed. These challenges present an opportunity for Zimbabwean researchers to contribute to the growing debate on ELSI of and development of appropriately tailored frameworks in line with various ongoing initiatives to build capacity for addressing and regulating current end emerging issues for biobanking, human genomics applications and data sharing in Africa.

## The Zimbabwe ELSI initiative for biobanking and genomics: GILES

**Figure 1.**
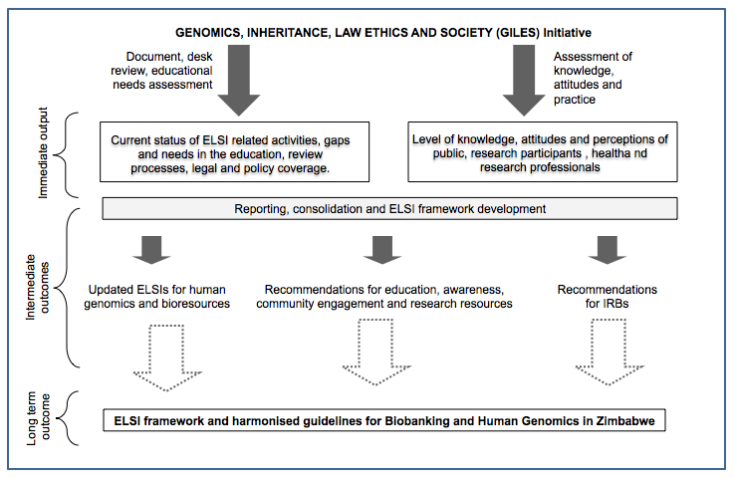
GILES initiative strategy.

Driven by the need to understand the current status and to determine needs for building capacity and harmonised guidelines for addressing ELSI of biobanking and human genomics in Zimbabwe, the Genomics Inheritance Law Ethics and Society (GILES) initiative was launched in 2015. The strategy involving steps to establish empirical evidence for ELSI regulations and knowledge-based participation in biobanking and human genomics for research and healthcare is shown in Figure 1 and the methods are outlined below. The rationale behind the methods, site selection and emerging findings are also summarized. Full accounts of the methods and results will be reported in separate manuscripts.

Prior to the development of data collection tools and fieldwork, the research team held meetings to determine key issues regarding biobanking and human genomics in Zimbabwe. The topics of genomics and biobanking, although commonplace, may appear daunting and too advanced among researchers in Zimbabwe due to various reasons including limitations in graduate and advanced training programmes. The interdisciplinary nature of ELSI research in topics which are cross-cutting in health, biomedicine and society, motivated a team of experts from diverse backgrounds was necessary and included bioethics, genomics, medicine, psychology, psychiatry, biomedical sciences and sociology. The majority of the GILES team were novices in the subjects of biobanking and genomics and associated ethical issues. Therefore, informational sessions led by the key investigators formed part of the project implementation strategy to ensure good understanding of the subject topics under study.

**Figure 2.**
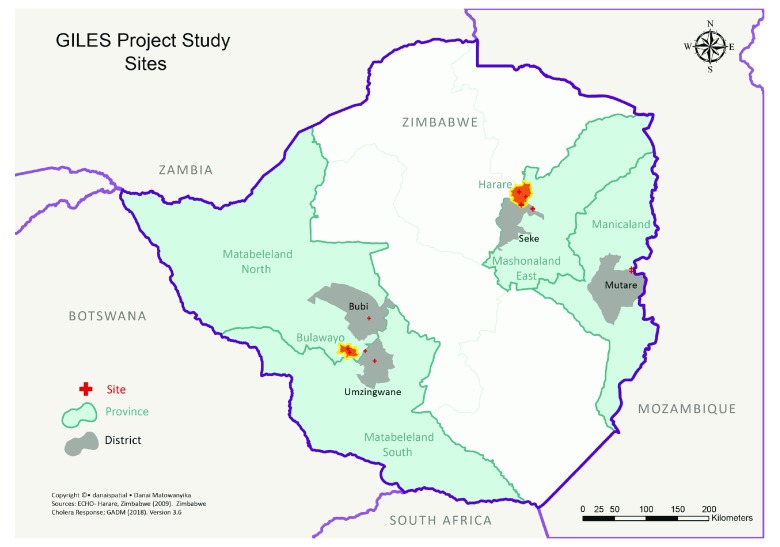
GILES project sites.

The GILES project employed a multi-methods approach, which included document reviews and an explorative qualitative study with targeted informant interviews and focus group discussions to understand the ELSIs and governance of biobanking and human genomics for health research and clinical applications in Zimbabwe. The qualitative method was used to establish subjective experiences of participants regarding biobanking and genomics as a basis for development of more focused studies and theoretical framework in future. The study was conducted in six provincial regions namely – Harare, Bulawayo, Mashonaland East, Manicaland, Matabeleland North and Matabeleland South ( Figure 2). The site selection was primarily based on the researchers’ affiliations in Harare and Mutare (Manicaland province). Historically, these regions have been favoured for clinical research work and represent the major ethnic group who speak the Shona language. Therefore, further considerations were made to include a wider ethnicity and geographical representation from the Matabeleland region. The Ndebele-speaking population are mostly located in Matabeleland North and Matabeleland South, with Bulawayo as the capital city. Populations in these regions are often under-represented in health research despite representing the second most populous ethnic group in Zimbabwe.

The health authorities are instrumental to accessing communities for research in Zimbabwe. We sought permission from the Ministry of Health and Child Care to engage with healthcare workers and members of the communities through local clinics and hospitals. Social scientists from the GILES project team guided the interviews and discussions.

### Analysis of ELSI regulations

With the fast pace of advanced technologies such as large scale biobanking, next generation sequencing and genomics, the current ethical review and regulatory structures may be inadequate in upholding ELSI requirements which ensure human subject protection while optimising research in Zimbabwe. To understand how ELSI are addressed and regulated in Zimbabwe and how they are used to govern biobanking and genomics for human health research and clinical applications, desk reviews of legal and policy documents, and regulatory instruments were conducted. In total, 76 documents were reviewed inclusive of the Zimbabwe Constitution, regulations, policies, national guidelines and guideline documents from institutions which collect biospecimens for research or clinical diagnostics use. Content analysis was used to determine the presence and absence of information or guidelines regarding the collection, storage, exportation and analysis of biological specimens and data, biospecimen and data sharing, data security and consent.

### Application of ELSI in research projects

All human subject research protocols are submitted for review, approval and registration through institutional and the national research ethics committee at the Medical Research Council of Zimbabwe (MRCZ). This provided a rich source of materials to analyse, as a proxy for the current practices among researchers in addressing ELSIs of biobanking and genomics by reviewing protocols, which involve human subjects and biospecimen collection/biobanking and genetics/genomics. In total, 200 protocols submitted to MRCZ for review from 2010 to 2016 were reviewed using a checklist. The 200 protocols were identified through the review of the Research Proposal register and electronic database maintained by MRCZ starting with recent submissions and moving backwards in time. At the time of the study, the register contained about 1600 entries. As this was the first time to conduct such a study, a preliminary assessment of research protocols by the ethics experts of the research team determined that there was poor consistency in the manner in which ELSIs were addressed in projects undertaking biospecimen collections and genetic or genomic analysis. Therefore a key objective in the GILES initiative was to generate empirical evidence to understand the needs for developing updated guidance for ELSI of human biobanking and genomics for researchers and ethics committees. We analysed content from the research protocols regarding ELSIs such as consent, privacy and confidentiality protections, community consultation and engagement, biorepositories (use, governance and security, specimen sharing and transfers), data sharing and security, informed consent features, descriptions of risks and benefits, long-term storage and implementation of sample disposal plans.

### Knowledge, attitudes and practice of biobanking and genomics

In Africa, there exist a wide range of perceptions about biobanking and processes such as sample collection and storage are surrounded in controversy, suspicion and other beliefs ^22, 23^. Ethical issues such as stigma, informed consent, privacy and confidentiality are emerging in the use and sharing of genetic information. A call for increased publications describing perceptions of the diverse African researchers, ethics committees and communities regarding genomics ^24, 25^, resonates on the key aim of the GILES project in characterising the broad range of knowledge, attitudes and perceptions about biobanking and genomics, and the rationales behind them. We targeted research scientists and healthcare workers who collect biospecimens which are used or have potential to be used for human genomics research or clinical diagnosis. The wider community represented prospective donors of biospecimen collection and participants in genetic or genomic analysis. The qualitative study approach was deemed appropriate at this exploratory stage. Applying a combination of in-depth interviews (IDIs) and focus group discussions (FGDs) allowed for an understanding of the meaning people give to their experiences particularly for terminologies, which may be less familiar generally. The research team held various workshops to develop consensus on the terminologies for use during the development of IDI and FGD guides. The process entailed generating ideas, recording the ideas, discussing the ideas, voting on the ideas, translation of items and triangulation. For effective communication with the individuals and communities around Zimbabwe, the guides were developed in English language and translated into local languages Shona and Ndebele. Topic guides and prompt statements were used to explore general issues about biobanking of biospecimens and their use in genomic analysis in healthcare and research. In-Depth Interviews were conducted among 31 individuals consisting of 3 spiritual and religious leaders, 11 researchers, 5 regulatory and ethics experts, 9 health service providers, 2 policymakers and 1 journalist. A total of 15 Focus Group Discussions were conducted among healthcare workers and community members from 6 provincial region ( Table 1). All IDIs and FGDs were audiotaped and transcribed then translated into English for analysis. Data processing and analysis was conducted using a combination of thematic and constant comparison analytical approaches. Complete reports of this process and the detailed results will be presented in a separate research manuscript.

**Table 1.** Number of focus group discussions participants by province.

Province	No. of participants by designation		TOTAL
	Healthcare workers	Community members	TOTAL
Harare	25	23	48
Mashonaland East		20	20
Bulawayo	35	24	59
Matebeleland South		14	14
Matebeleland North		9	9
Manicaland	20	19	39
TOTAL	80	109	189

Community engagement and education are key to fulfilling ethical requirements by promoting understanding of complex subjects such as biobanking and genomics. The H3Africa consortium researchers are actively incorporating such strategies into their research programs ^26, 27^. The participation of the various stakeholders in the GILES project has facilitated a first step towards community engagement in biobanking and genomics in Zimbabwe. This has created a foundation, which may be useful for future research and capacity building programmes tailored for the diverse local and regional communities.

## Lessons learnt and opportunities for the GILES initiative

Lessons learnt during during implementation:

- Due to the complex nature of the topics under study, the GILES project offered a learning opportunity to the research team members who were novices in the topics of biobanking and genomics.
- For a wide geographical spread, the process of getting support letters and approvals from the relevant government departments, local authorities and other universities was mired in bureaucracy, slowing down project momentum and timelines. Although there was enthusiasm about the need to conduct such research, educative discussions with the heads of authorities and institutions, were key for obtaining approvals.
- Since this was the first time such a study was being implemented, it was necessary to have back and forth meetings during the protocol development process, particularly for refining the methodology, tools and translations into the two main local languages to ensure consistency of terms and concepts.
- Use of well established community advisory boards was also key for engaging critical stakeholders.
- Religious and traditional views have a huge influence on communities’ perceptions on participation in biobanking and genomics research
- Development of terminology for biobanking, genomics and ethics, may have benefited from wider consultation among various stakeholders prior to conducting the study.
- A generous amount of time was necessary for field-work especially in mobilization of participants, to ensure wide population coverage.

Opportunities

- Development of ELSI research focused on biobanking and genomics is needed to further understand specific needs among the various professional and community groups.
- To develop educational material to improve awareness and participation in genomic research particularly and health research in general.
- To apply community engagement strategies to develop appropriate terminology and improved understanding of biobanking and genomics for use in research and health dialogue. This has potential to build relationships, increase trust, improve consent processes and empower local communities ^28^.
- To develop targeted community engagement interventions based on established beliefs, perceptions and practices.
- To develop updated guidelines and policies to guide research scientists and ethics committees.
- To strengthen capacity among research ethics committee members and regulatory authorities.

## Conclusions and next steps

The GILES initiative established a platform for the study of ELSI related to bio- and data resources for human genomics activities, which involve biospecimen collection, storage, analysis, data sharing and use (biobanking, databases and bioinformatics) in Zimbabwe. This was achieved through a multi-disciplinary approach involving research scientists, health and academic professionals and community members. The GILES initiative is innovative in being the first to address ELSI regarding the human genomics resources for health research and application in Zimbabwe. This was enriched by using a comprehensive methodology encompassing desk reviews and interviews and by involving a diverse research team of biomedical scientists, clinicians, public health and ethics experts and social scientists. Further comprehensive descriptions of findings will be reported separately.

Zimbabwe is a country that is experiencing growth in genomic research and biobanking and yet ELSI of human bioresources and genomics are inadequately applied and poorly understood. Growing interest in the application of genomics in medicine and diagnostics implies that there is a need for a paradigm shift in the education and training of researchers, health professionals and the public on ELSI of biobanking and human genomics. The GILES initiative will culminate in strengthening capacity through education, training and community engagement. We envisage the use of local beliefs, perceptions and folklore in developing tools, which can provide more efficient means for research participant recruitment, awareness and consent processes for biobanking and genomics research. Capacity building will empower students, faculty and health professionals, researchers, regulatory authorities, public health scientists and the wider public. In the future, workshops will be conducted to develop recommendations, which will be availed to institutional review boards, research ethics committees, regulatory bodies and government in order to tailor the ELSI framework which protects and empowers research participants, researchers and health professionals while advancing biobanking and human genomics in Zimbabwe and the African region.

Anecdoctally, there is limited understanding of ELSI implications for genomic research and healthcare in Zimbabwe, a situation which may apply across the continent. The experiences in implementation of the GILES initiative and preliminary observations suggest a need for more thorough localised ELSI research projects in Africa to accommodate the diversity of cultural norms and levels of capacity in use of biobanking and human genomics technologies. This reiterates calls for the development of more tailored national and regional guidelines, which support the inevitable and growing nature of collaborative biobanking and genomics research ^7, 13^. The GILES initiative presents an example, which may be used to conduct such explorative work in other African countries. It is also among a select few studies in Zimbabwe and Africa, which have employed an inclusive approach for exploring the needs for future development of an evidence-based ELSI framework. This will provide opportunities for education, community engagement and capacity building for tailored ethical frameworks appropriate for African communities.

## Disclaimer

The views expressed in this article are those of the author(s). Publication in AAS Open Research does not imply endorsement by the AAS.

## Ethical considerations

The GILES study was approved by the Joint Research Ethics Committee (JREC) at Parirenyatwa Central Hospital, and College of Health Sciences at University of Zimbabwe Reference: number 06/15. Additionally, the study was reviewed and approved by the national research ethics Committee at the Medical Research Council of Zimbabwe Reference number: MRCZ/A/2051). Prior to the focus group discussions and in-depth interviews, written informed consent was obtained from all participants. All participants were given pseudonyms to use instead of their names for confidentiality purposes. Permissions to conduct interviews and document reviews were obtained from the heads of the relevant institutes and organisations. Permission to engage with community members and health care staff was obtained from the Ministry of Health and Child Care as we used health centres to recruit community participants. Further permission was obtained from the community leaders from the respective communities.

## Data availability

No data is associated with this article.
