# Supplementary material for: Understanding Ethical, Legal and Societal Issues (ELSIs) in Human Biobanking and Genomics for Research and Healthcare in Zimbabwe: The Genomics Inheritance Law Ethics and Society (GILES) initiative
Source: AAS Open Res. 2019 Jun 12;2:1. Originally published 2019 Jan 8. [Version 2] doi: 10.12688/aasopenres.12917.2 (PMC7194152; doi:10.12688/aasopenres.12917.2)
Supplement: Peer review history

# Peer review: Reviewer response for version 2

**DOI:** 10.21956/aasopenres.14052.r26982

**Published:** 2019-06-17

**Authors:** Erisa Sabakaki Mwaka

**Affiliations:** 1 Department of Human Anatomy, College of Health Sciences, Makerere University, Kampala, Uganda

First, I would like to thank you for the opportunity of reviewing this paper. The authors have addressed the comments that were requested of them to my satisfaction. I recommend that the manuscript be indexed.

I confirm that I have read this submission and believe that I have an appropriate level of expertise to confirm that it is of an acceptable scientific standard.

# Peer review: Reviewer response for version 2

**DOI:** 10.21956/aasopenres.14052.r26983

**Published:** 2019-06-12

**Authors:** Francis Masiye

**Affiliations:** 1 Centre for Medical Ethics and Law, Department of Medicine, Faculty of Medicine & Health Sciences, Stellenbosch University, Cape Town, South Africa

Thanks for giving me an opportunity to review both the revised article and the author responses to the issues that were raised during the initial review. I have reviewed both the revised article and author responses. The authors have responded adequately to the issues I had raised during the initial review. Therefore, I am happy to approve this version of the article for indexing.

 Best wishes,

 Francis.

I confirm that I have read this submission and believe that I have an appropriate level of expertise to confirm that it is of an acceptable scientific standard.

# Peer review: Reviewer response for version 1

**DOI:** 10.21956/aasopenres.13987.r26746

**Published:** 2019-02-08

**Authors:** Francis Masiye

**Affiliations:** 1 Centre for Medical Ethics and Law, Department of Medicine, Faculty of Medicine & Health Sciences, Stellenbosch University, Cape Town, South Africa

This manuscript presents interesting and novel primary data about views and opinions of research participants interviewed in a qualitative study nested within the Genomics Inheritance Law, Ethics and Society (GILES) initiative in Zimbabwe. The study appears to have been well designed and executed, but additional details about the structure of the manuscript, which should include the following order: background/introduction, objectives, methods, results/findings, discussion of the findings, recommendations and conclusions are needed. Additionally, this manuscript should have been classified as a research article and not as an open letter since it reports on findings of the qualitative study.

 This manuscript has much to teach the bio-baking and genomics research community, but would benefit from revisions as follows:

1. The objectives of the empirical study should be articulated in the manuscript.
2. The methods section should explain how the qualitative data from the in-depth interviews (IDIs) and Focus Group Discussions (FGDs) was processed and analyzed.
3. The results section could benefit from a substantial organizational overhaul with an eye towards enhancing the clarity of the central message of the manuscript including direct quotations from research participants. Finally, enhancing the emphasis on key study findings that are novel, exciting, and have direct implications for addressing ethical, legal and societal issues (ELSI) for bio-banking and genomics in Africa  is important.

I confirm that I have read this submission and believe that I have an appropriate level of expertise to confirm that it is of an acceptable scientific standard, however I have significant reservations, as outlined above.

# Response

**Published:** 2019-05-19

**Authors:** Alice Matimba

**Affiliations:** Wellcome Genome Campus Advanced Courses and Scientific Conferences, UK

Thank you for reviewing our article. We have addressed the comments as indicated in bold below. This includes clarification of the objectives of the article as an Open letter providing an overview of the GILES initiative. More detailed manuscripts for the various results of the project are currently underway.

**Reviewer 2**

This manuscript presents interesting and novel primary data about views and opinions of research participants interviewed in a qualitative study nested within the Genomics Inheritance Law, Ethics and Society (GILES) initiative in Zimbabwe. The study appears to have been well designed and executed, but additional details about the structure of the manuscript, which should include the following order: background/introduction, objectives, methods, results/findings, discussion of the findings, recommendations and conclusions are needed. Additionally, this manuscript should have been classified as a research article and not as an open letter since it reports on findings of the qualitative study. **–** **The objectives of the article have been clarified to fit with expectations for the classification.**

This manuscript has much to teach the bio-baking and genomics research community, but would benefit from revisions as follows:

1.      The objectives of the empirical study should be articulated in the manuscript. **– This has been revised to emphasise the main aim of the paper as well as the main aims of the GILES Initiative.**

2.      The methods section should explain how the qualitative data from the in-depth interviews (IDIs) and Focus Group Discussions (FGDs) was processed and analyzed **. ******– As indicated in the main aim of the paper which was to describe the strategy and experiences of the GILES initiative. Detailed reports and results will be published in separate research manuscripts which will also provide more details on methods including management of qualitative data.**

3.      The results section could benefit from a substantial organizational overhaul with an eye towards enhancing the clarity of the central message of the manuscript including direct quotations from research participants. Finally, enhancing the emphasis on key study findings that are novel, exciting, and have direct implications for addressing ethical, legal and societal issues (ELSI) for bio-banking and genomics in Africa is important. **– In line with the above the paper has been revised to emphasise its main aim in providing an overview of the strategy of the project, and experiences of the researchers. Detailed reports and results will be published in separate research manuscripts which will also provide more details on the study methods adopted for the different components of the GILES study.**

# Peer review: Reviewer response for version 1

**DOI:** 10.21956/aasopenres.13987.r26714

**Published:** 2019-01-14

**Authors:** Erisa Sabakaki Mwaka

**Affiliations:** 1 Department of Human Anatomy, College of Health Sciences, Makerere University, Kampala, Uganda

Overall, the presentation of this paper is a bit confusing.

1. In the abstract it is stated that the paper “ *describes the strategy undertaken for the development and implementation of the GILES project and discusses the importance of such an initiative for characterisation of ELSI of human biobanking and genomics in Zimbabwe and Africa*”. However the way it is written  is more inclined towards reporting the experiences of the GILES project.
2. “ *The GILES project employed a multi-methods approach, which included document reviews and an explorative qualitative study with targeted informant interviews and focus group discussions to understand the ELSIs and governance of biobanking and human genomics for health research and clinical applications in Zimbabwe”.* But it seems the paper is presenting more of the experiences of the GILES project than an explorative qualitative study. The voice of the respondents is conspicuously missing. I think the main problem is with the rationale of this paper. I suggest that the introduction be revised and the precise objective of this paper be clearly articulated. Otherwise it is confusing in its current form.
3.  “ *ELSI regulations”; “There exist gaps in the current guidelines and policies for addressing ELSI of biobanking and genomics in Zimbabwe”*. Later on in the same paragraph the authors state *“Content analysis will determine the presence and absence of information or guidelines regarding the collection, storage, exportation and analysis of biological specimens and data, biospecimen and data sharing, data security and consent.”* It sounds like the content analysis is not yet done. Then, what is this paper reporting? Desk review?
4. Desk review of “legal and policy documents, and regulatory instruments” is vague. It is indicated that protocols were also reviewed. Which protocols? Protocols of which research? For which time period; since MRCZ was established? Etc. It is thus not clear which exact documents from MRCZ were reviewed. The documents should be explicitly stated and references cited where applicable.
5. The first paragraph of the “ *Knowledge, attitudes and practice of ELSI of biobanking and genomics”* section is presented in present tense; is it a preamble? If so, was it from literature? Provide reference citation. Are these study findings?  
6. “ *Application of ELSI in research projects*” section indicates that FGDs and in-depth interviews were conducted however, how this was done is not clearly articulated. The last paragraph of this section talks about community engagement and there seems to be a disconnect with the preceding sections. Please include a bridging statement.
7. At this point it difficult to tell whether this paper is presenting the experiences of the GILES project or perceptions and views of individual participants? It is a bit confusing to the reader.
8. Let this paper be revised to present the results of the desk review and experiences of the GILES project.
9. “ *The limited understanding of ELSI applications for genomic research and healthcare in Zimbabwe is a situation, which also applies across the continent”.* It is not clear whether this statement is derived from the results or it’s from literature?

I confirm that I have read this submission and believe that I have an appropriate level of expertise to confirm that it is of an acceptable scientific standard, however I have significant reservations, as outlined above.

# Response

**Published:** 2019-05-19

**Authors:** Alice Matimba

**Affiliations:** Wellcome Genome Campus Advanced Courses and Scientific Conferences, UK

Thank you for reviewing our article. We have addressed the comments as indicated in bold below. This includes clarification of the objectives of the article as an Open letter providing an overview of the GILES initiative. More detailed manuscripts for the various results of the project are currently underway.

**Reviewer 1**

Overall, the presentation of this paper is a bit confusing.

1.      In the abstract it is stated that the paper “ *describes the strategy undertaken for the development and implementation of the GILES project and discusses the importance of such an initiative for characterisation of ELSI of human biobanking and genomics in Zimbabwe and Africa*”. However the way it is written is more inclined towards reporting the experiences of the GILES project. **–** **This has been well noted and the abstract has been refined and inclined towards reporting of the strategy undertaken and experiences of the project.**

2.      “ *The GILES project employed a multi-methods approach, which included document reviews and an explorative qualitative study with targeted informant interviews and focus group discussions to understand the ELSIs and governance of biobanking and human genomics for health research and clinical applications in Zimbabwe”.* But it seems the paper is presenting more of the experiences of the GILES project than an explorative qualitative study. The voice of the respondents is conspicuously missing. I think the main problem is with the rationale of this paper. I suggest that the introduction be revised and the precise objective of this paper be clearly articulated. Otherwise it is confusing in its current form. **–** **The objective of the paper has been revised both in abstract and in the Introduction. We have also indicated that the key aim was to describe the strategy and that detailed methodology of the various activities and their results will be reported in separate research manuscripts.**

3.       “ *ELSI regulations”; “There exist gaps in the current guidelines and policies for addressing ELSI of biobanking and genomics in Zimbabwe”*. Later on in the same paragraph the authors state *“Content analysis will determine the presence and absence of information or guidelines regarding the collection, storage, exportation and analysis of biological specimens and data, biospecimen and data sharing, data security and consent.”* It sounds like the content analysis is not yet done. Then, what is this paper reporting? Desk review? **–** **This paragraph has been updated. As indicated above this manuscript summarises the methods and more details will be obtained in the full manuscripts which are underway.**

4.      Desk review of “legal and policy documents, and regulatory instruments” is vague. It is indicated that protocols were also reviewed. Which protocols? Protocols of which research? For which time period; since MRCZ was established? Etc. It is thus not clear which exact documents from MRCZ were reviewed. The documents should be explicitly stated and references cited where applicable. **–** **As indicated above this manuscript summarises the methods and more details will be obtained in the full manuscripts which are underway. We have however provided clarification regarding the protocols reviewed.**

5.      The first paragraph of the “ *Knowledge, attitudes and practice of ELSI of biobanking and genomics”* section is presented in present tense; is it a preamble? If so, was it from literature? Provide reference citation. Are these study findings? **–** **This has been revised and references provided.**

6.      “ *Application of ELSI in research projects*” section indicates that FGDs and in-depth interviews were conducted however, how this was done is not clearly articulated. The last paragraph of this section talks about community engagement and there seems to be a disconnect with the preceding sections. Please include a bridging statement. **– This section has been revised to reflect the objective of the paper which aimed at providing an overview of the project, summarise activities undertaken and their rationale.**

7.      At this point it difficult to tell whether this paper is presenting the experiences of the GILES project or perceptions and views of individual participants? It is a bit confusing to the reader. **–** **The former is correct. The revisions have highlighted the main aim of the paper describing strategy taken for the project, focusing on rationale and experiences of the research team.**

8.      Let this paper be revised to present the results of the desk review and experiences of the GILES project. **– Please refer to points above reading the main aim of the paper at this stage. As an open letter, the paper does not take the format of a research article. – Paper has been revised accordingly in line with above comments.**

9.      “ *The limited understanding of ELSI applications for genomic research and healthcare in Zimbabwe is a situation, which also applies across the continent”. *It is not clear whether this statement is derived from the results or it’s from literature? **– This sentence has been revised. The point is that more ELSI research is required and also more understanding and awareness about ethical issues in these advanced technologies is needed. This applies across all regions of the African continent where these technologies are only starting to be applied, and each country would need to tailor interventions appropriate to their settings.**
